# Clinical Characteristics and Outcomes of Patients Admitted with Brief Resolved Unexplained Events to a Tertiary Care Pediatric Intensive Care Unit

**DOI:** 10.7759/cureus.8664

**Published:** 2020-06-17

**Authors:** Ali A Alhaboob

**Affiliations:** 1 Pediatrics, King Saud University, Riyadh, SAU

**Keywords:** brief resolved unexplained events, life threatening events, diagnosis, mortality

## Abstract

The aim of this study was to determine outcomes of patients admitted to a tertiary care pediatric intensive care unit (PICU) with brief, resolved, unexplained event (BRUE), and to review the diagnostic and treatment options utilized for such patients. A retrospective data analysis was conducted for infants and children who were admitted to the PICU at a tertiary hospital with a diagnosis of BRUE over a period of three years (2015-2017). The study included 30 infants, 15 males, and 15 females. All patients survived to hospital discharge. The most frequent presenting symptoms and signs were apnea (73.3%), cyanosis (60.0%), and cough (20.0%). The most frequent reported affected systems were respiratory (33.3%), gastrointestinal (20%), and infection-related illness (20.0%). We conclude that the careful history taking, complete physical examination, and the appropriate workup for patients with BRUE play an integral role in optimum health service and utilization of critical care beds. Survival to hospital discharge with no serious in-hospital events warrants the adaptation of evidence-based medicine guidelines to stratify such patients based on the risk of recurrence or a serious underlying condition. Prospective multicenter studies are recommended to explore the effectiveness of such guidelines implementation on outcomes and diagnostic testing in such patients to optimize the utilization of the limited critical care beds.

## Introduction

An apparent life-threatening event (ALTE) is defined as "an episode that is frightening to the observer and is characterized by some combination of apnea, color change, a marked change in muscle tone, and choking or gagging". In clinical practice, the original definition of ALTE was found difficult to apply. These difficulties are mainly due to the fact that under the ALTE definition, the majority of patients are asymptomatic and the diagnosis and management of symptomatic infants (e.g., those presenting with fever and respiratory distress) should be differentiated from asymptomatic patients. The second challenge is that the reported symptoms under the ALTE definition frightening to caregivers are not really life-threatening as it is mostly benign manifestations of normal infant physiology or self-limited conditions [1]. 

Given the challenges that clinicians faced in applying the term ALTE in clinical practice, the American Academy of Pediatrics recently addressed the need for a new definition and classification of ALTE [1,2]. They recommended the replacement of the term ALTE with a new term, brief resolved unexplained event (BRUE), stratifying infants based on the risk of recurrence of a serious underlying condition, and providing evidence-based recommendations for infants at lower-risk [3].

According to a previous report, BRUE account for 0.6%-0.8% of all emergency room visits for infants younger than one year [4]. However, the true prevalence of BRUE is unknown, and it is estimated that 0.5%-0.6% of healthy term infants have experienced BRUE [5]. About half of these infants are subsequently diagnosed with an underlying condition that can explain the occurrence of BRUE while the cause remains unknown in the other half [5]. In a healthy-looking infant, risk factors for severe underlying pathology include prematurity, possible child abuse, seizures, and recurrent BRUE [4].

Clinicians are faced with difficulties in evaluating infants presenting with BRUE, who typically require basic cardiopulmonary resuscitation. Another challenge that clinicians face is evaluating and managing infants with BRUE who were initially hospitalized for reasons other than BRUE.

This study aims to determine the characteristics and outcomes of patients admitted to the pediatric intensive care unit (PICU) with BRUE and to review their diagnostic and management approaches.

## Materials and methods

This retrospective data analysis included all patients less than one-year-old who were admitted with the diagnosis of BRUE to the PICU of King Khalid University Hospital, Riyadh, Saudi Arabia between 2014 and 2016. During this period, 1455 patients were admitted to the PICU, of which 30 (2.1%) were admitted with a diagnosis of BRUE. The diagnosis was based on thorough history taking and complete physical examination by the attending physician. Their medical records were reviewed for their demographic characteristics (gestational age, gender, and post-natal age upon admission to the PICU) as well as their presenting symptoms and signs (fever, cough, shortness of breath, cyanosis, apnea, vomiting/regurgitation, convulsions, bradycardia, and hypoglycemia). We also collected diagnostic workup data, including results of a complete blood count, serum electrolytes, liver function tests, blood gas analysis, blood cultures, nasopharyngeal aspirate for virology (NPA), and, whenever applicable, a metabolic screen. Other collected data included results of chest X-ray (CXR), electrocardiogram (ECG), Holter monitoring, echocardiography, esophageal pH monitoring, and electroencephalogram (EEG). Furthermore, different therapies that those patients received were also collected. Moreover, outcome data including the length of stay at the PICU, survival discharge from PICU, and any associated comorbidity. The study was approved by the Institutional Review Board of the College of Medicine, King Saud University.

Statistical analysis

The data were analyzed using the Statistical Package for the Social Sciences, version 23 (SPSS Inc., IBM, New York, US). A t-test was used to analyze continuous variables; the results are expressed as median and inter-quartile (IQ). Conversely, the chi-square test was used to analyze categorical variables; the results are presented as frequencies and percentages.

## Results

Table 1 shows the characteristics of studied patients. The study included 30 infants, 15 males, and 15 females. They were 15 preterms (gestational age < 36 weeks) and 15 full-term (gestational age ≥ 36 weeks) babies. Upon admission to the PICU, their postnatal ages were: 18 (60%) < one month old, 8 (26.6%) were one month to < three months old, 2 (6.7%) were three-six months old, and two (6.7%) were ≥ six months to < 12 months. The most frequent presenting symptoms and signs among studied patients were apnea (73.3%), followed by cyanosis (60%). The other reported symptoms and signs are shown in Table 1.

**Table 1:** Characteristics of studied patients (N = 30) PICU: Pediatric intensive care unit * A patient might have more than one symptom and sign. ** A patient might have more than one diagnostic workup.

Characteristics	Number	Percentage
Gender		
Male	15	50%
Female	15	50%
Gestational age		
Preterm (≤ 37 weeks)	15	50%
Full term (> 38 weeks)	15	50%
Post-natal age on admission		
< 1 month	18	60%
1 month to < 3 months	8	26.6%
3-6 months	2	6.7%
≥ 6 months - < 12 months	2	6.7%
Presenting symptoms and signs^*^		
Fever	2	6.7%
Cough	6	20%
Shortness of breath	2	6.7%
Cyanosis	18	60%
Apnea	22	73.3%
Vomiting/regurgitation	1	3.3%
Convulsions	4	13.3%
Bradycardia	4	13.3%
Hypoglycemia	1	3.3%
Diagnostic workup^**^		
Blood culture	21	70%
Nasopharyngeal aspirate for virology studies	10	33.3%
Chest X-ray (CXR)	6	20%
Electrocardiogram (ECG)	1	3.3%
Holter monitoring	1	3.3%
Echocardiography	6	20%
Esophageal pH monitoring	2	6.7%
Electroencephalogram (EEG)	5	16.7%
Diagnosis by system affected		
Neurological	1	3.3%
Cardiovascular	1	3.3%
Respiratory	5	16.7%
Gastrointestinal	5	16.7%
Infectious disease	5	16.7%
Metabolic disease	2	6.7%
Undiagnosed	11	36.7%
Outcome:		
Length of stay at PICU (Days) (Median) (IQR) Survival PICU discharge	5.5 (1.25, 8) 30	100%

Blood culture was the most frequent ordered investigation where it was done for 21 patients (70%), followed by NPA virology where it was done for 10 patients (33.3%). On the other hand, electrocardiogram and Holter monitoring were done for one patient for each (3.3%). The diagnosis was not specified in 11 (36.7%) patients. On the other hand, lower respiratory tract infection, gastroesophageal reflux disease, and infectious diseases (suspected sepsis) were reported in five patients (16.7%) for each, while the metabolic disease was reported in two cases (6.7%). Cardiovascular and neurological disease (suspected seizure disorder) were reported in one patient (3.3%) for each. The median length of stay was 5.5 days (interquartile range, 1.25-8 days) and all the patients survived till PICU discharge and there was no reported mortality and/or morbidity upon hospital discharge.

**Figure 1:**
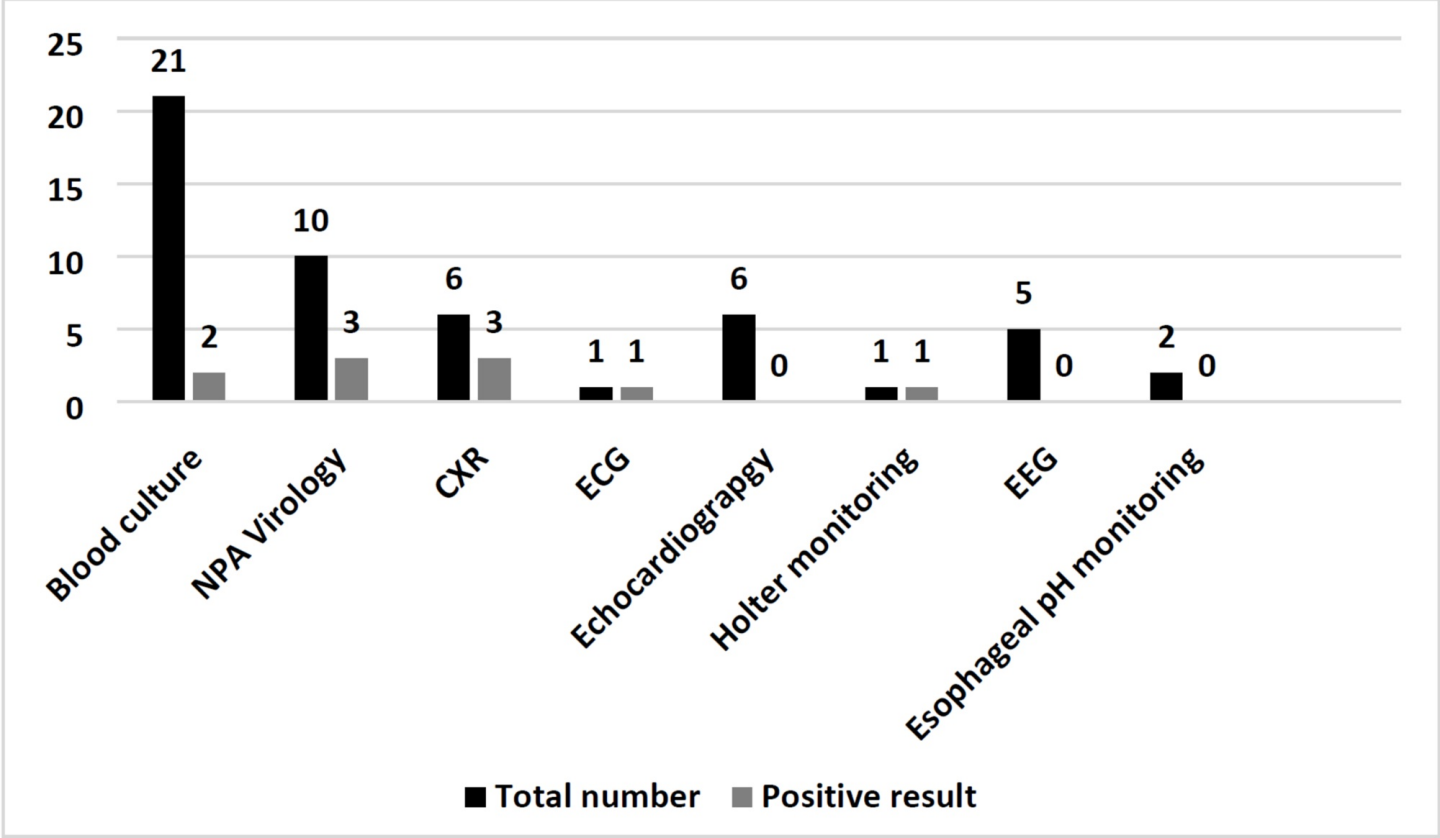
Frequency of diagnostic workup and positive results among the patients NPA: Nasopharyngeal aspirate; CXR: Chest X-ray; ECG: Electrocardiogram; EEG: Electroencephalogram.

Figure 1 shows the frequency of diagnostic workup and positive results among the patients. Positive blood culture was reported in two out of the twenty-one patients who had a blood culture done. NPA virology was positive in three out of the ten patients who had this test done. Other diagnostic workup included positive findings in the chest X-rays of three out of six patients who had a chest X-ray, normal echocardiography findings in the six patients who had echocardiography, normal EEG in the five patients who had an EEG, and normal esophageal pH probe monitoring in the two patients who had this test. On the other hand, ECG and Holter monitoring showed positive findings in one patient who had such a test.

**Figure 2:**
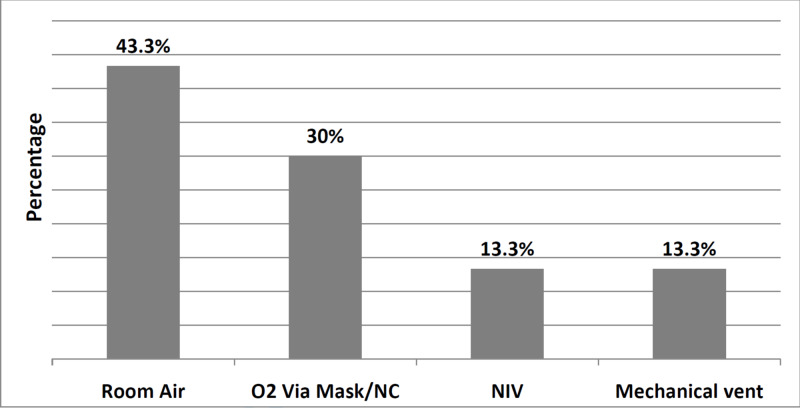
Frequency for modalities of respiratory therapy provided to the patients O2: Oxygen; NC: Nasal cannula; NIV: Non-invasive ventilation; Vent: ventilation

Figure 2 shows the relative frequency for modalities of respiratory therapy provided to the studied patients. Nine patients (30%) received oxygen by nasal cannula/face mask, four (13.3%) received non-invasive ventilation, and another four (13.3%) received conventional mechanical ventilation.

Table 2 shows the frequency of presenting symptoms, signs, diagnosis, and treatment interventions among preterm and full-term patients.

The median gestational age was 34 (27, 36) weeks in preterm infants and 36 (36, 44) weeks in term babies. The most frequent presenting symptoms among preterm and term patients were apnea (12 versus 10), convulsions (4 versus 0), and bradycardia (3 versus 1). On the other hand, full-term infants presented more with cough (5 versus 1), shortness of breath (2 versus 0), cyanosis 10 versus 8), vomiting/regurgitation (1 versus 0), and hypoglycemia (1 versus 0) when compared to pre-term patients. The affected systems and provided treatment for both groups were similar or negligible differences. The average length of stay in the PICU was 6.1 (1, 17) days in preterm and 6.5 (1, 26) days in full-term patients, respectively.

**Table 2:** Frequency of presenting symptoms, signs, diagnosis, and treatment interventions among preterm and full-term patients PICU: Pediatric intensive care unit * A patient might have received more than one treatment intervention. ** Respiratory therapies involved oxygen via nasal cannula or face mask, non-invasive ventilation, and conventional mechanical ventilation *** One patient with a cardiovascular diagnosis of long QT syndrome was referred to the cardiac center.

Variables	Preterm (N = 15)	Full term (N = 15)
Gestational age (Weeks):		
Median (Min, Max)	34 (27,36)	36 (36,44)
Symptoms and signs		
Fever	1 (6.7%)	1 (6.7%)
Cough	1 (6.7%)	5 (33.3%)
Shortness of breath	0	2 (13.3%)
Cyanosis	8 (53.3%)	10 (66.7%)
Apnea	12 (80%)	10 (66.7%)
Vomiting/regurgitation	0	1 (6.7%)
Convulsions	4 (26.7%)	0
Bradycardia	3 (20%)	1 (6.7%)
Hypoglycemia	0	1 (6.7%)
Diagnosis by affected system		
Neurological	1 (6.7%)	0
Cardiovascular	0	1 (6.7%)
Respiratory	3 (20%)	2 (13.3%)
Gastrointestinal	3 (20%)	2 (13.3%)
Infectious disease	2 (13.3%)	3 (20%)
Metabolic disease	1 (6.7%)	1 (6.7%)
Undiagnosed	5 (33.3%)	6 (40%)
Treatment interventions^*^		
Antibiotics	10 (66.7%)	9 (60%)
H2 blockers	4 (26.7%)	5 (33.3%)
Prokinetic medication	2 (13.3%)	2 (13.3%)
Respiratory therapy^** ^	10 (66.7%)	11 (73.3%)
Other treatment^***^	0	1 (6.7%)
Length of stay at the PICU (days)		
Median (Min, Max)	6.1 (1,17)	6.5 (1,26)

## Discussion

The major indication for the hospitalization of infants with BRUE (ALTE) is to assess those patients for a possible underlying serious disease and/or life-threatening event that they might encounter if not hospitalized. However, few studies have described outcomes in infants who were hospitalized with a diagnosis of BRUE [6-9].

In the current study, out of 1455 admissions during a three-year period, we reported 30 patients admitted with a diagnosis of BRUE, representing 2.06% of the total admissions. Such frequency in the current study is similar to European reports, where the frequency of BRUE was in the range of 0.6 to 5 per 1000 live births and another report showed a frequency of 2.46 per 1000 live births [10-12]. These variations among different studies might be attributed to the differences in the definition of BRUE and whether this diagnosis was based on the family or caregiver perception or physician evaluation. In the current study, the diagnosis was made by physician evaluation rather than family description and/or perception. Another possible factor that might have influenced our results was that all of our patients were recruited from the emergency department upon emergency physician consultation to PICU. This might have caused an underestimation of the actual number of patients who presented to our institute with BRUE because not all of such patients are being consulted and/or admitted to the PICU.

The current study showed an equal frequency of BRUE among premature and full-term infants. Moreover, BRUE was more frequent among those who were younger than one month old. These findings are consistent with those of other reports which showed the highest prevalence of BRUE during the neonatal period or in infants younger than three months [13-16]. However, some investigators reported a higher incidence of BRUE among premature babies while other authors reported a lower incidence [17-21].

Apnea and cyanosis were reported as the most frequent presenting symptom among the patients which comes in agreement with other reports [22].

Patients with BRUE usually undergo several investigations to reach a possible diagnosis. In the current study, blood culture was the most frequently ordered investigation but it was reported to be positive in two patients only which is similar to other reports [23]. Moreover, the current study is similar to others in reporting the respiratory tract infections and gastrointestinal tract disorders to be a more common diagnosis among patients with BRUE [23,24]. On the other hand and in contrast to other studies, where seizure disorder and possible neurological disease are common in patients with BRUE, only one patient in the current study had a neurological disorder [23-25]. However, 11/30 of studied patients (36.7%) no diagnosis could be reached. A possible explanation may be due to the overestimation of parents for their baby symptoms and signs, especially in the case of a first baby. Another possible explanation in the case of formulating the diagnosis of BRUE based on the parents' or caregiver perception rather than good history taking and complete physical examination by the physician. Moreover, patients who might be hospitalized with a diagnosis of BRUE just have a normal physiologic variable in such an age group.

The current study showed favorable outcomes among patients with BRUE, with no reported mortality and/or morbidity. This is similar to other studies conducted on ALTE infants [26-28]. Moreover, some reports followed such patients up to the age of 18 months and others followed such patients up to the age of three years and both reported no mortality and/or morbidity, ensuring the favorable outcome of such patients [27,28]. However, a mortality rate as high as 34.7% among such patients has been reported [9].

In general, some factors determine the outcome of children who experience BRUE. Mortality rates reported to be higher and outcomes are less favorable in children with a serious underlying medical condition including a disorder characterized by seizures or another neurologic condition [29]. Poor outcomes have also been reported among some BRUE patients, who eventually died of sudden infant death syndrome [5].

Previous studies have attempted to identify infants at high-risk and criteria, such as age less than or equal to one month and/or multiple episodes of ALTE, could correctly predict children at risk for severe ALTE requiring admission [30]. Another report identified prematurity as a risk factor of severe ALTE [14].

Limitations

Our study has some limitations, including its retrospective design. Additionally, the small sample size does not permit us to draw relevant conclusions, and the study design can be viewed as a pilot study, that does not permit us to observe causality. While our study portrays a tertiary center experience with BRUE over three years, future studies with multicenter involvement can further define the association of antenatal factors and BRUE.

## Conclusions

BRUE represent a diverse disorder. Careful history taking, complete physical examination, and appropriate workup might play an integral role in optimum health service and utilization of critical care beds. Survival to hospital discharge in low-risk infants, with no serious in-hospital events, warrants adaptation of evidence-based clinical practice guidelines to stratify infants at risk. Prospective, multi-center studies are recommended to explore the effectiveness of such guidelines' implementation on patients' outcomes and resource utilization.
